# Onyxis

**Published:** 1842-11-26

**Authors:** 


*Onyxis.*—The diseased lateral growth of the nail of the great toe, when it presses into the surrounding soft parts, and causes a fungous granulation to spring forth, is nearly as painful as the operation usually adopted for its cure. M. Payan, the principal surgeon of the Hotel-Dieu, at Aix, in an essay on caustics, which recently gained the prize offered by the proprietors of the "Bulletin de Thérapeutique," advises the application of the Vienna paste to that portion of the matrix of the nail, which corresponds to the part involved in the vicious growth. The adjacent parts are to be protected by adhesive plaster. By the destruction of the matrix, the reproduction of the diseased nail is prevented. The Vienna paste is the potassa cum calce, prepared with six parts of quick lime and five of pure potass.—*Prov. Med. Journ.*, Oct. 22, 1842.

